# Comparison of 2-octyl cyanoacrylate and *n*-octyl cyanoacrylate topical skin adhesives for wound closure after ankle fracture surgery: a prospective randomized trial

**DOI:** 10.1038/s41598-023-43471-6

**Published:** 2023-10-10

**Authors:** Young Hwan Park, Sei Wook Son, Sang Roc Han, Hak Jun Kim

**Affiliations:** 1 Department of Orthopedic Surgery, Korea University Guro Hospital, 148 Gurodong-ro, Guro-gu, Seoul, 08308 Republic of Korea; 2 Department of Orthopedic Surgery, Korea University Medical Center, Seoul, Republic of Korea

**Keywords:** Health care, Medical research

## Abstract

To date, only a few clinical studies have investigated the differences between 2-octyl cyanoacrylate and *n*-octyl cyanoacrylate topical skin adhesives (TSAs). This study aimed to compare the outcomes of the two TSAs for wound closure after ankle fracture surgeries. Fifty-six patients were randomized to receive either a 2-octyl or *n*-octyl cyanoacrylate TSA. At 3 and 6 months after surgery, wound cosmetic outcomes were assessed using the Hollander Wound Evaluation Scale (HWES), and patient satisfaction for wound cosmesis was assessed using the visual analog scale (VAS) and 5-item Likert scale. Functional outcomes at 6 months after surgery were assessed using the Olerud–Molander Ankle Score (OMAS). Fifty-five patients completed the study protocol. Within the follow-up period, no differences were found between the two groups in terms of HWES, VAS, 5-item Likert scale, and OMAS. 2-octyl cyanoacrylate TSA and *n*-octyl cyanoacrylate TSA were comparable options for wound closure after ankle fracture surgeries in terms of wound cosmesis, patient satisfaction, and functional outcome.

## Introduction

Cyanoacrylates, formed by the condensation of cyanoacetate and formaldehyde, were initially synthesized in 1949 and have been used since as a topical skin adhesive (TSA) in wound closure^1^. They immediately polymerize upon exposure to the skin and form a clean, strong adhesive bond that holds together the wound corners, enabling normal wound healing under the film^2^. With advantages in scar cosmesis, infection prevention, easy and rapid use, and painless removal, cyanoacrylate TSAs are recently replacing traditional wound closure methods, such as skin stapling and suturing^3–9^.

Among several types of cyanoacrylate, 2-octyl cyanoacrylate and *n*-butyl cyanoacrylate are representative materials for medical applications^2^. They are manufactured as commercial TSAs and have been used according to clinician preference. Animal studies have reported that *n*-butyl cyanoacrylate TSA showed lower adhesion strength, wound-bursting strength, and adhesive flexibility than 2-octyl cyanoacrylate TSA^10–12^, whereas in clinical studies, *n*-butyl cyanoacrylate TSA showed similar cosmetic outcomes to 2-octyl cyanoacrylate TSA^13,14^. These results suggest that the inferior physical properties of *n*-butyl cyanoacrylate have little effect on clinical performance. However, since the wound size was small (< 4 cm) in both previous clinical studies and one study lacked a validated wound assessment tool^13^, further studies are needed to establish clear conclusions.

Therefore, we determined to investigate whether *n*-butyl cyanoacrylate TSA is comparable to 2-octyl cyanoacrylate TSA for orthopedic surgeries. This study aimed to prospectively compare *n*-butyl cyanoacrylate and 2-octyl cyanoacrylate TSAs for wound closure after ankle fracture surgeries using a validated wound assessment tool.

## Patients and methods

### Study design

This study was designed as a prospective randomized controlled trial comparing 2-octyl cyanoacrylate TSA and *n*-octyl cyanoacrylate TSA for wound closure after ankle fracture surgeries using a 1:1 randomization. After approval by the local ethics committee (Korea University Guro Hospital Institutional Review Board, 2018GR0099), the study was registered in the Clinical Research Information Service (https://cris.nih.go.kr) database, which is a non-profit online registration system for clinical trials established by the Korea Disease Control and Prevention Agency^15^. All study protocols were conducted in accordance with the Consolidated Standards of Reporting Trials guidelines^16^.

### Inclusion/exclusion criteria

Patients who required open reduction and plate fixation for isolated lateral malleolar fractures were included. Among the patients with rotational ankle fractures, those with associated posterior malleolar fractures or a partial deltoid ligament rupture but only required open reduction and plate fixation for the lateral malleolar fracture were also included. The exclusion criteria were as follows: (1) < 18 or > 65 years of age; (2) previous surgeries at the ankle joint; (3) significant medical comorbidities potentially affecting wound healing (e.g., end-stage renal failure, peripheral vascular disease)^17,18^, (4) history of keloid formation, open fracture, or other notable skin injuries around the ankle joint combined with fracture; (5) blistering on the incision site; (6) > 10% of ankle edema in the injured side compared to the uninjured side measured by the figure-of-eight-20 method^19^, (7) workers’ compensation, and (8) participation in another study. Among the eligible patients, those who provided informed consent for participation were finally included.

### Participants

**Figure 1:**
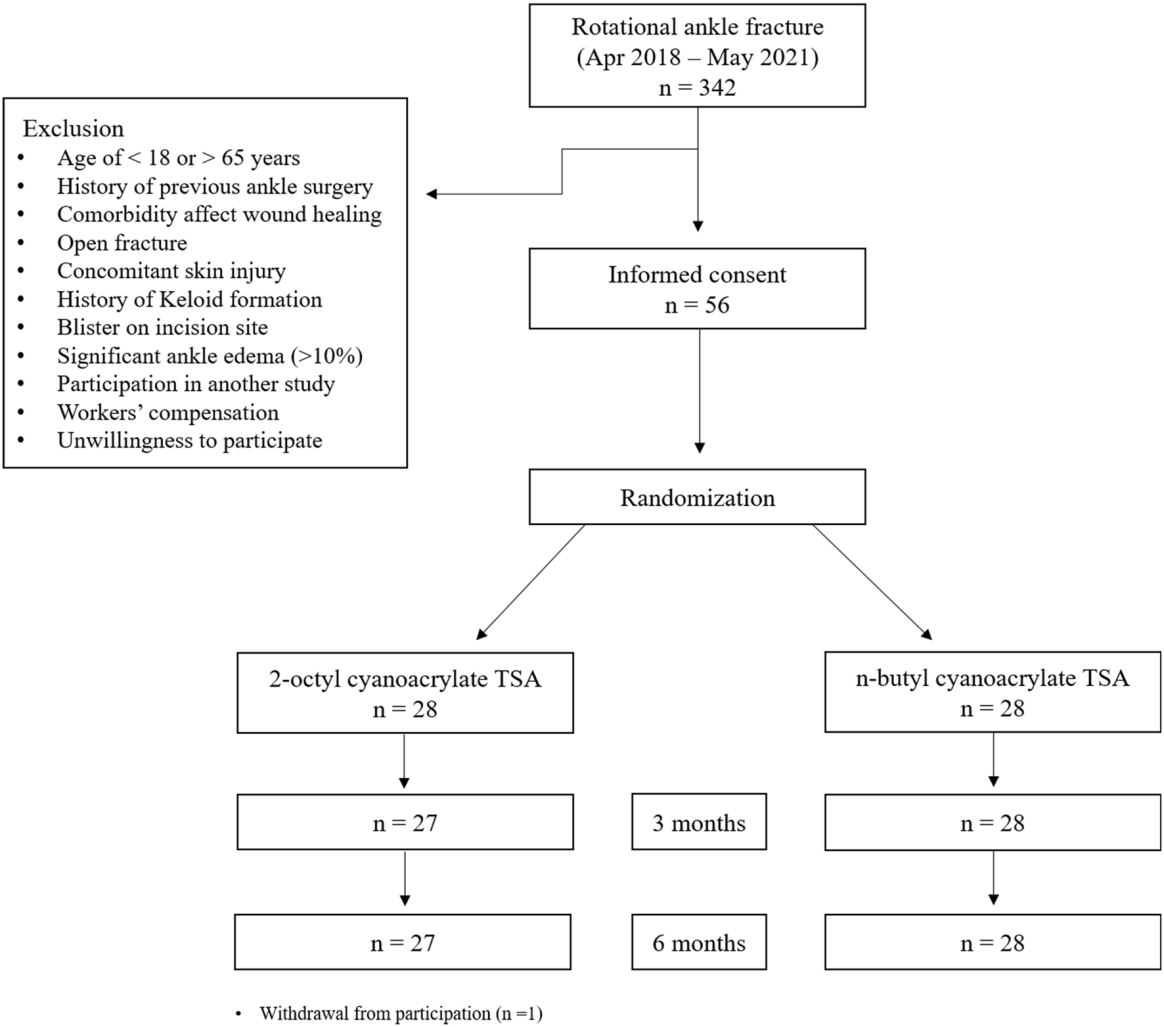
Flow diagram of the study. *TSA* topical skin adhesive.

Between April 2018 and May 2021, 342 patients visited our institution for rotational ankle fracture, and 78 patients met the inclusion/exclusion criteria. Of these patients, 22 were excluded because they did not wish to participate in the study, leaving 56 patients randomized into both groups (Fig. 1). The full date of the first registration was 20/08/2021, and the registration number was KCT0006476.

### Randomization

Patients were randomly assigned to one of the two groups using sequentially numbered opaque-sealed envelopes made by the study coordinator. The randomization sequence was constructed using computer software (Excel 2016; Microsoft, Redmond, WA, USA)^20^. Neither the patients nor the surgeons knew the randomization outcomes until the day of surgery.

### Preoperative assessment

Preoperatively, 3 to 7 days of bed rest and elevation of the injured ankle were performed to reduce swelling to an acceptable level for internal fixation. To quantify preoperative soft tissue swelling at the incision site, both ankles were measured using the figure-of-eight-20 method^19^. The percentage of measured edema on the injured side was calculated as a fraction of the edema of the injured side to that of the uninjured side, and this value was used to identify intergroup differences.

### Interventions

Open reduction and plate fixation of the fractures were performed by a single surgeon on the basis of standard AO principles^21^. Closure of the fascial and subcuticular skin layers was performed in the same way in both groups using 2-0 and 3-0 synthetic absorbable braided sutures (Vicryl Plus, Ethicon Ltd, Edinburgh, UK). Either 2-octyl cyanoacrylate TSA (Dermabond Prineo, Ethicon, Somerville, NJ, USA) or *n*-butyl cyanoacrylate TSA (LiquiBand, Advanced Medical Solutions, Winsford, UK) was then applied in two thin layers with a drying time of 30–60 s between layers to enable polymerization. In the 2-octyl cyanoacrylate group, this process was performed over the contained polyester mesh according to the manufacturer’s instructions. A standard absorbent dressing was then applied and changed on the second day after surgery. Polyester mesh in the 2-octyl cyanoacrylate group was removed 14 days after surgery, as per the manufacturer’s instructions.

### Primary and secondary outcomes

As a validated wound assessment tool, the Hollander Wound Evaluation Scale (HWES) was used as a primary outcome measure. The HWES consists of five subscales, and the wounds with a score of 6 were considered to have an optimal cosmetic appearance: step-off of borders (edges not on the same plane; 0 for yes, 1 for no), contour irregularities (wrinkled skin near the wound; 0 for yes, 1 for no), margin separation (gap between side; 0 for yes, 1 for no), edge inversion (wound not properly everted; 0 for yes, 1 for no), excessive distortion (swelling/edema/infection; 0 for yes, 1 for no), and overall appearance (subject appearance: 0 for poor, 1 for acceptable)^22,23^. The assessment of HWES was independently performed by two orthopedic surgeons, and in cases of disagreement between them, the final decision was made via consensus.

Secondary outcome measures included the visual analog scale (VAS) and 5-item Likert scale to assess patient satisfaction with wound cosmesis. The VAS score was determined by measuring the distance (mm) on the 10-cm line between the “worst possible scar” anchor and the patient mark, providing a range of scores from 0 to 100^24,25^. On a 5-point Likert scale, patients were asked about their satisfaction with wound cosmesis in one of the following items: very unsatisfied, unsatisfied, neutral, satisfied, and very satisfied^26^. The HWES, VAS, and 5-point Likert scale were assessed at 3 and 6 months of follow-up visits. To reduce the bias of outcome due to the incision length, the incision lengths were measured to investigate whether there was a difference between the two groups.

Additionally, the Olerud–Molander Ankle Score (OMAS; scale, 0–100 points) was assessed at 6-month follow-up visits to measure the functional outcome. A higher OMAS indicates better outcomes and fewer symptoms^27^.

### Blinding

A single-blinded study design was used. Surgeons and patients were not blinded to group allocation. Outcome assessments and statistical analyses were performed by one author who did not participate in the operative procedures and was blinded to the allocations.

### Sample size calculation

The non-inferiority trial method was used to determine the appropriate sample size^28^. Using the HWES at 6 months postoperatively as the primary outcome of measure, the sample size was calculated to have a power of 80% and a significance level of 0.05. To determine whether the 2-octyl cyanoacrylate TSA was not inferior to the n-butyl cyanoacrylate TSA, 25 patients per group were required to have 80% power that the lower limit of a one-sided 95% confidence interval for the difference between two treatments would be above the non-inferiority margin of −1.0 point (standard deviation, 1.25 points). Since there was no universally established minimal clinically important difference for the HWES, the non-inferiority margin was selected by investigators on the basis of a previously conducted prospective study^29^. Adding an assumed dropout rate of 10%, 28 patients were finally enrolled in each group.

### Statistical analysis

The Kolmogoro–Smirnov test was used to determine data normality. The independent t-test or Mann–Whitney U test was used to compare the following continuous variables: age, body mass index (BMI), HWES score, VAS score, OMAS, and incision length. The chi-squared test or Fisher’s exact test was used to compare the following categorical variables: sex, smoking status, diabetes, injury mechanism, and 5-point Likert scale score. Inter-observer reliability for the HWES was assessed by calculating the kappa coefficient (κ), and the values were judged as follows: poor (< 0.0), slight (0.01–0.20), fair (0.21–0.40), moderate (0.41–0.60), substantial (0.61–0.80), and almost perfect (0.81–1.00)^30^. All statistical analyses were performed using SPSS (version 23.0; IBM Corp, Armonk, NY), with statistical significance set at p < 0.05.

## Results

### Patient characteristics

Fifty-five patients completed 6 months of follow-up. There were no intergroup differences in terms of age, sex, BMI, smoking status, diabetes, injury mechanism, ankle edema, or incision length (Table 1).

**Table 1:** Patient characteristics.

	2-Octyl cyanoacrylate	*n*-Butyl cyanoacrylate	p-value
	(n = 27)	(n = 28)	
Age, years	46.6 ± 14.8	45.9 ± 14.8	0.862
Sex			0.282
Female	13	18	
Male	14	10	
BMI, kg/m^2^	25.5 ± 3.9	23.8 ± 2.6	0.259
Smoking status			0.861
Smoker	5	4	
Previous smoker	1	1	
Nonsmoker	21	23	
Diabetes			0.611
Type II	2	1	
None	25	27	
Injury mechanism			0.484
Fall	19	20	
Sports	6	3	
MVA	2	4	
Others	0	1	
Ankle edema, %	106.6 ± 2.6	106.2 ± 2.7	0.643
Incision length, cm	9.8 ± 2.2	9.0 ± 2.0	0.143

Values are presented as means ± standard deviations or number of patients.

*BMI* body mass index, *MVA* motor vehicle accident.

### Primary outcome analysis

**Figure 2:**
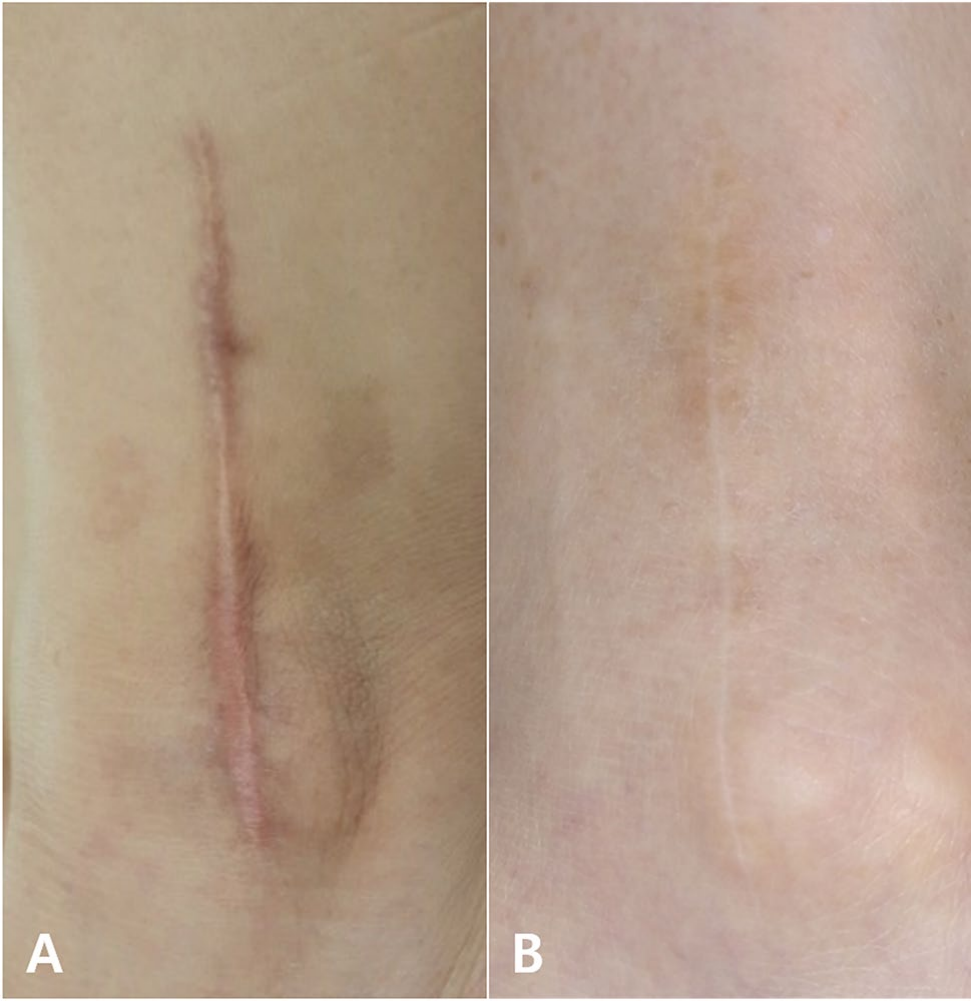
Clinical photograph of wounds treated with *n*-butyl cyanoacrylate topical skin adhesive. (**A**) Incision scar with poor overall appearance due to step-off borders, margin separation, and excessive distortion. [Hollander Wound Evaluation Scale (HWES), 1 point]. (**B**) Incision scar with acceptable overall appearance (HWES, 6 points).

No statistically significant differences were found between the two groups in the total and individual HWES scores (Table 2 and Fig. 2). The HWES scores assessed by the two observers had almost perfect reliability (κ = 0.822).

**Table 2:** Hollander wound evaluation scale.

	3 months			6 months		
	2-Octyl cyanoacrylate (n = 27)	*n*-Butyl cyanoacrylate (n = 28)	p-value	2-Octyl cyanoacrylate (n = 27)	*n*-Butyl cyanoacrylate (n = 28)	p-value
Step-off of borders	0.9 ± 0.3	0.9 ± 0.3	0.617	0.9 ± 0.4	0.9 ± 0.3	0.371
Contour irregularities	0.7 ± 0.4	0.8 ± 0.4	0.939	0.7 ± 0.4	0.8 ± 0.4	0.478
Margin separation	0.6 ± 0.5	0.6 ± 0.5	0.708	0.6 ± 0.5	0.6 ± 0.5	0.708
Edge inversion	1.0 ± 0.2	0.9 ± 0.3	0.583	0.9 ± 0.3	0.9 ± 0.3	0.971
Excessive distortion	0.7 ± 0.5	0.6 ± 0.5	0.317	0.8 ± 0.4	0.7 ± 0.5	0.390
Overall appearance	0.6 ± 0.5	0.8 ± 0.4	0.343	0.7 ± 0.4	0.7 ± 0.4	0.939
Total score	4.5 ± 1.3	4.6 ± 0.9	0.856	4.7 ± 1.3	4.8 ± 1.0	0.700

Values are presented as means ± standard deviations.

### Secondary outcome analysis

**Figure 3:**
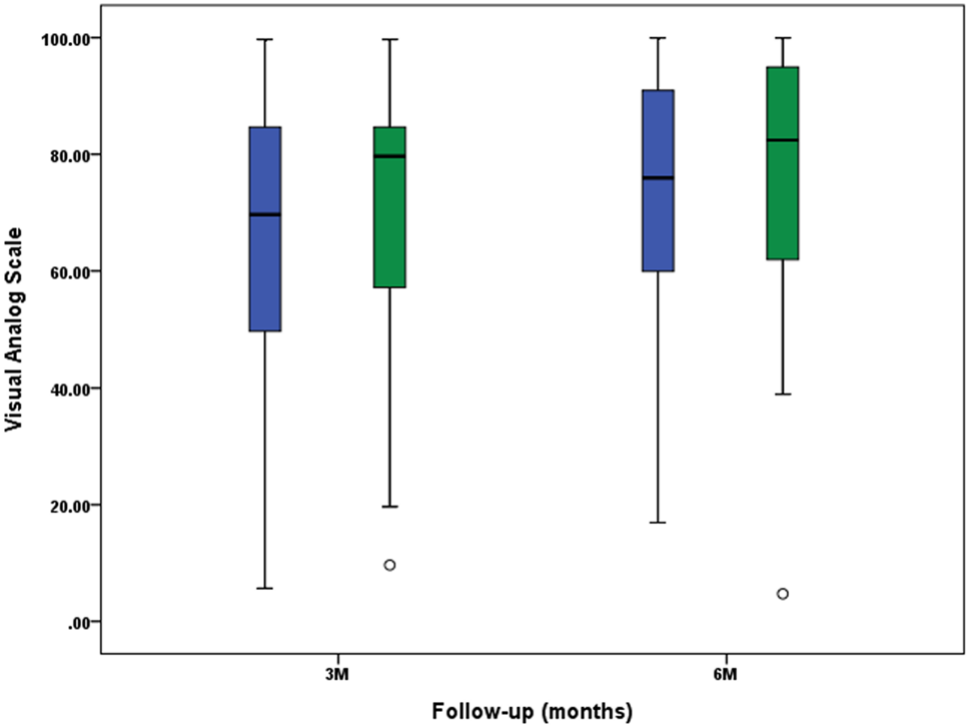
Visual analog scale scores for patient satisfaction to wound cosmesis. No significant difference was found between the 2-octyl cyanoacrylate TSA (blue) and *n*-octyl cyanoacrylate TSA (green) groups at 3 and 6 months after surgery (p = 0.659 and p = 0.700, respectively). TSA, topical skin adhesive.

**Figure 4:**
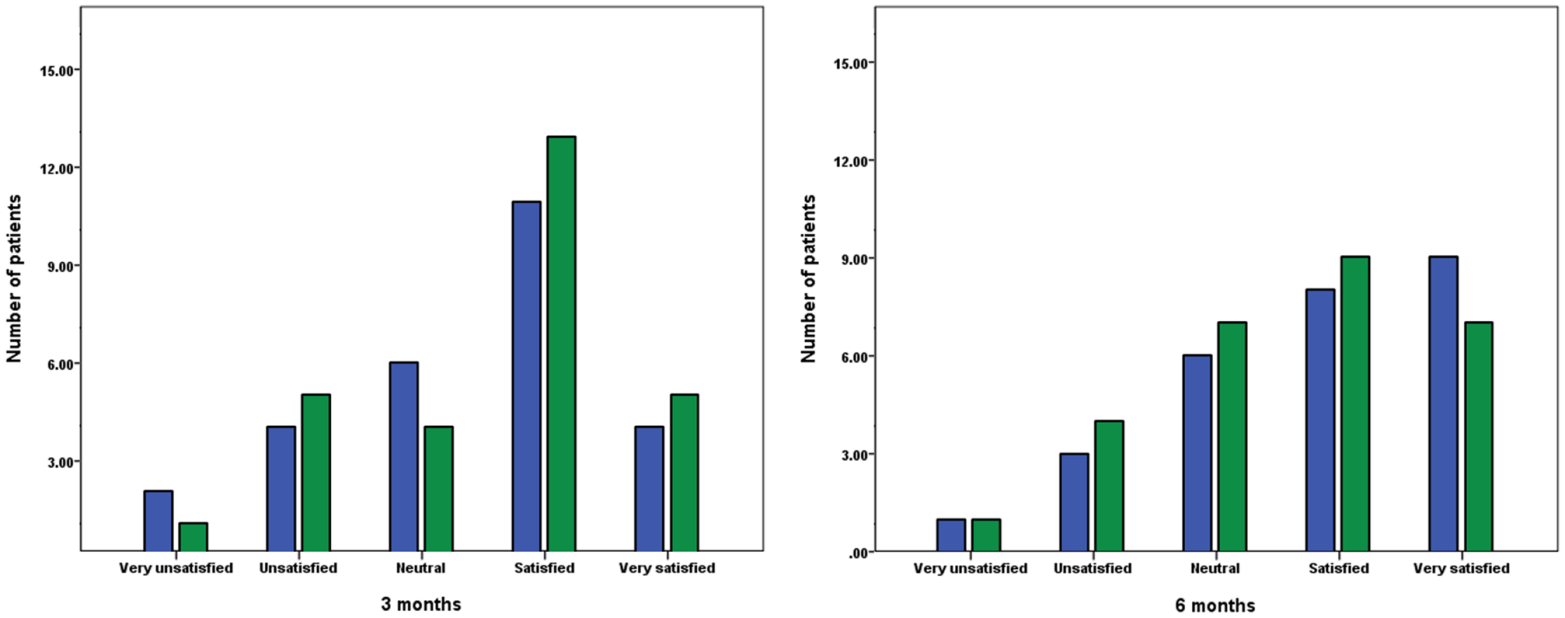
5-point Likert scale scores for patient satisfaction to wound cosmesis. No significant difference was found between the 2-octyl cyanoacrylate TSA (blue) and *n*-octyl cyanoacrylate TSA (green) groups at 3 and 6 months after surgery (p = 0.915 and p = 0.971, respectively). *TSA* topical skin adhesive.

The VAS and 5-point Likert scale scores for patient satisfaction with wound cosmesis did not show any statistically significant differences between the two groups at 3 and 6 months after surgery (Figs. 3 and 4). The 6-month postoperative OMAS was 73.6 ± 16.5 and 72.9 ± 14.5 in the 2-octyl cyanoacrylate and *n*-butyl cyanoacrylate groups, respectively, with no statistically significant difference (p = 0.864).

### Complications

Except for one patient in the 2-octyl cyanoacrylate group who had a superficial wound infection by methicillin-sensitive *Staphylococcus aureus*, no postoperative complications were reported. The patient was treated with oral antibiotics and repeated dressing, and the wound healed without sequelae.

### Costs

The 2-octyl cyanoacrylate and *n*-butyl cyanoacrylate TSAs cost $124.53/unit (3.6 ml/unit, $3.46/0.1 ml) and $68.50/unit (0.8 ml/unit, $8.56/0.1 ml), respectively. One unit of skin adhesive was used for wound closure in each patient. These costs vary according to product volume, region, and country.

## Discussion

Despite the increasing use of 2-octyl and *n*-butyl cyanoacrylate TSAs for wound closure in orthopedic surgeries, comparative studies on clinical outcomes after using both materials are limited. We found no difference between the two materials in terms of wound cosmesis, patient satisfaction, and functional outcome. Despite showing inferior mechanical properties, the clinical outcomes of *n*-butyl cyanoacrylate TSA are not inferior to those of 2-octyl cyanoacrylate TSA. Therefore, clinicians may freely choose either material, according to their preference, the wound size, and cost difference.

Although several animal studies demonstrated clear differences in the mechanical properties between the 2-octyl cyanoacrylate TSA and the *n*-butyl cyanoacrylate TSA, there were no differences in the clinical outcomes evaluated through this study. To explain this discrepancy, we deduced the following two possibilities. First, unlike animal studies that evaluated the wound bursting strength and tensile strength immediately after closure, the cosmetic wound assessments in this study were performed at least 3 months after surgery. This time interval may be sufficient for scar remodeling, diluting the cosmetic differences caused by the mechanical properties of the two materials. Second, various stretching forces acting on the incision site of the lateral malleolus in this study may have been below the threshold that could reflect the difference between the mechanical properties of the two materials. To clarify these possibilities, future studies should be conducted in the early phases of surgery with incision sites that are more vulnerable to tensile forces, such as total knee arthroplasty.

The strengths of this study are that it was designed prospectively, used a validated wound assessment tool, and applied TSAs to wound closure in fracture surgery. In our previous study^13^, we retrospectively compared the outcomes of 2-octyl cyanoacrylate TSA, *n*-butyl cyanoacrylate TSA, and nylon sutures for wound closure in Achilles tendon rupture repair surgery, and found no differences in patient satisfaction and complications between the two cyanoacrylate TSAs. However, the validity of the outcome measure was questioned. In addition, via a prospective randomized clinical trial, Osmond et al.^14^ compared 2-octyl cyanoacrylate TSA and *n*-butyl cyanoacrylate TSA. They used the HWES to assess wound cosmesis and found no intergroup differences. However, applying these results to orthopedic surgery was limited because the patient cohort was confined to children with facial lacerations of < 4 cm. Therefore, we believe that this study will have a high clinical utility by complementing previous studies.

This study had two limitations. First, the LiquiBand used for the *n*-butyl cyanoacrylate TSA group was a blend of 90% *n*-butyl cyanoacrylate and 10% 2-octyl cyanoacrylate. Compared to the 2-octyl cyanoacrylate TSA group, in which 100% 2-octyl cyanoacrylate was used, using 10% 2-octyl cyanoacrylate in the *n*-butyl cyanoacrylate TSA group may have biased the results of the study. We decided to use LiquiBand because there was no commercially available monomeric n-butyl cyanoacrylate TSA product at the time of the study in our country. However, considering the mechanical study that reported no differences in wound bursting strength and tensile strength between LiquiBand and monomeric *n*-butyl cyanoacrylate TSA (Histoacryl)^12^, we believe that the use of LiquiBand would not significantly affect the results. Second, the incidence of complications was not compared. Since the complication rates following the application of TSAs were low^31,32^, comparing the incidence of complications will likely show no statistically significant differences between the groups, whether a true difference exists (i.e., a type 2 error). Therefore, considering the sample size of this study, we did not compare the incidence of complications.

In conclusion, of *n*-butyl cyanoacrylate TSA for wound closure after ankle fracture surgeries was not inferior to 2-octyl cyanoacrylate TSA in terms of wound cosmesis, patient satisfaction, and functional outcome. These findings suggest that, like 2-octyl cyanoacrylate TSA, *n*-butyl cyanoacrylate TSA is a viable alternative to traditional wound closure methods in orthopedic surgery.

## Data Availability

All data supporting our findings are contained within the manuscript. All data in this study are freely available to any researcher for noncommercial purposes.

## Abbreviations

TSA: Topical skin adhesives
HWES: Hollander Wound Evaluation Scale
VAS: Visual analog scale
OMAS: Olerud–Molander Ankle Score
BMI: Body mass index

## References

[1] Coover HW, Joyner FB, Sheare TH, Wicker TH (1959). Chemistry and performance of cyanoacrylate adhesives. J. Soc. Plast. Eng..

[2] Singer AJ, Quinn JV, Hollander JE (2008). The cyanoacrylate topical skin adhesives. Am. J. Emerg. Med..

[3] Park YH, Song JH, Choi GW, Kim HJ (2018). Comparison of 2-octyl cyanoacrylate topical skin adhesive and simple interrupted nylon sutures for wound closure in ankle fracture surgery. Foot Ankle Int..

[4] Dowson CC, Gilliam AD, Speake WJ, Lobo DN, Beckingham IJ (2006). A prospective, randomized controlled trial comparing *n*-butyl cyanoacrylate tissue adhesive (LiquiBand) with sutures for skin closure after laparoscopic general surgical procedures. Surg. Laparosc. Endosc. Percutan. Tech..

[5] Lin M, Coates WC, Lewis RJ (2004). Tissue adhesive skills study: The physician learning curve. Pediatr. Emerg. Care.

[6] Quinn J (1997). A randomized trial comparing octylcyanoacrylate tissue adhesive and sutures in the management of lacerations. JAMA.

[7] Spauwen PH, de Laat WA, Hartman EH (2006). Octyl-2-cyanoacrylate tissue glue (Dermabond) versus Monocryl 6 x 0 sutures in lip closure. Cleft Palate Craniofac. J..

[8] Scott GR, Carson CL, Borah GL (2007). Dermabond skin closures for bilateral reduction mammaplasties: A review of 255 consecutive cases. Plast. Reconstr. Surg..

[9] Singer AJ, Kinariwala M, Lirov R, Thode HC (2010). Patterns of use of topical skin adhesives in the emergency department. Acad. Emerg. Med..

[10] Singer AJ, Perry LC, Allen RL (2008). In vivo study of wound bursting strength and compliance of topical skin adhesives. Acad. Emerg. Med..

[11] Singer AJ (2004). Comparison of wound-bursting strengths and surface characteristics of FDA-approved tissue adhesives for skin closure. J. Adhes. Sci. Technol..

[12] Singer AJ, Perry L (2012). A comparative study of the surgically relevant mechanical characteristics of the topical skin adhesives. Acad. Emerg. Med..

[13] Park YH, Chang AS, Choi GW, Kim HJ (2018). A comparison of three methods of skin closure following repair of Achilles tendon rupture. Injury.

[14] Osmond MH, Quinn JV, Sutcliffe T, Jarmuske M, Klassen TP (1999). A randomized, clinical trial comparing butylcyanoacrylate with octylcyanoacrylate in the management of selected pediatric facial lacerations. Acad. Emerg. Med..

[15] Choi EK, Kim MJ, Lim NK, Park HY (2016). Review of the registration in the clinical research information service. J. Korean Med. Sci..

[16] Moher D (2010). CONSORT 2010 explanation and elaboration: Updated guidelines for reporting parallel group randomised trials. BMJ.

[17] Chiriano J (2010). Management of lower extremity wounds in patients with peripheral arterial disease: A stratified conservative approach. Ann. Vasc. Surg..

[18] Maroz N, Simman R (2013). Wound healing in patients with impaired kidney function. J. Am. Coll. Clin. Wound Spec..

[19] Rohner-Spengler M, Mannion AF, Babst R (2007). Reliability and minimal detectable change for the figure-of-eight-20 method of, measurement of ankle edema. J. Orthop. Sports Phys. Ther..

[20] Kim J, Shin W (2014). How to do random allocation (randomization). Clin. Orthop. Surg..

[21] Ruedi, T., Buckley, R. & Moran, C. *AO Principles of Fracture Management. Second Expanded Edition*. Vol 2. 871–897 (AO Publishing**,** 2007).

[22] Dumville, J.C. *et al.* Tissue adhesives for closure of surgical incisions. *Cochrane Database Syst. Rev*. CD004287 (2014). 10.1002/14651858.CD004287.pub4 PMC10074547 25431843

[23] Hollander JE, Singer AJ, Valentine S, Henry MC (1995). Wound registry: Development and validation. Ann. Emerg. Med..

[24] Quinn JV, Wells GA (1998). An assessment of clinical wound evaluation scales. Acad. Emerg. Med..

[25] Paul-Dauphin A, Guillemin F, Virion JM, Briancon S (1999). Bias and precision in visual analogue scales: A randomized controlled trial. Am. J. Epidemiol..

[26] Arrigoni SC, Halbersma WB, Grandjean JG, Mariani MA (2012). Patients' satisfaction and wound-site complications after radial artery harvesting for coronary artery bypass. Interact. Cardiovasc. Thorac. Surg..

[27] Olerud C, Molander H (1984). A scoring scale for symptom evaluation after ankle fracture. Arch. Orthop. Trauma Surg..

[28] Chow S, Shao J, Wang H (2003). Sample Size Calculations in Clinical Research, 57–59.

[29] Lewis, T.L. *et al.* Randomized controlled trial of topical skin adhesive vs nylon sutures for incision closure in forefoot surgery. *Foot Ankle Int*. 10711007211002501 (2021). 10.1177/10711007211002501 33870760

[30] Landis JR, Koch GG (1977). The measurement of observer agreement for categorical data. Biometrics.

[31] Chalmers BP (2017). Characterizing the diagnosis and treatment of allergic contact dermatitis to 2-octyl cyanoacrylate used for skin closure in elective orthopedic surgery. J. Arthroplasty.

[32] Michalowitz A, Comrie R, Nicholas C, Wagner M, Kehoe J (2020). Wound complications after 2-octyl skin closure systems for total joint arthroplasty. J. Bone Jt. Infect..

